# Methodology for reliable and reproducible cryopreservation of human cervical tissue

**DOI:** 10.1016/j.cryobiol.2017.06.004

**Published:** 2017-08

**Authors:** James M. Fox, Rebecca C. Wiggins, John W.J. Moore, Christine Brewer, Alison C. Andrew, Fabiola Martin

**Affiliations:** a Centre for Immunology and Infection, Department of Biology and Hull York Medical School, University of York, UK; b York Teaching Hospital, NHS Foundation Trust, York, UK

**Keywords:** *Ex vivo*, Cervix, Cervical tissue, Cryopreservation, Freezing, Thawing, Storage, Bio-banking

## Abstract

**Background:**

In order to conduct laboratory studies on donated cervical tissue at suitable times an effective and reliable cryopreservation protocol for cervical tissue is required.

**Methods:**

An active freezing approach was devised utilising 10% dimethyl sulfoxide in foetal bovine serum as a cryoprotective agent with a cooling rate of 1 °C/min to −50 °C then 10 °C/min to −120 °C; a related thawing protocol was also optimised which would allow for the bio-banking of cervical tissue. Viability of freshly harvested cervical tissue was compared to frozen-thawed samples utilising colorimetric MTT assay. In parallel, fresh and freeze-thawed samples were cultured and tested on days 1, 7 and 14 to determine whether bio-banking had detrimental effects on tissue viability over time.

**Results:**

Repeat testing revealed that tissue viability between fresh and freeze-thawed samples was comparable at all four time points (days 0, 1, 7 and 14) with no apparent reductions of viability, thus demonstrating this method of cryopreserving cervical tissue is reliable and reproducible, without detrimental effects on live tissue culture. We believe this methodology creates the opportunity for bio-banking donated cervical tissues, which aids improved experimental design and reduces time pressures and wastage.

## 1 Introduction

Extensive scientific literature has reported the utilisation of cervical tissue organo-typical models to further our understanding of HIV transmission and to aid development of potential therapeutic interventions [12], [15]. We are also aware of cervical explants being utilised to study mucosal epithelial cell differentiation and leukocyte infiltration [10], cytomegalovirus infection studies [6] and for vaccine research [2]. Almost exclusively, these studies and others were undertaken in fresh tissue transported without delay from hospitals to scientific laboratories for immediate use in experimental procedures. We aimed to conduct Human T-lymphotropic virus (HTLV)-1 *ex vivo* infection studies in donated cervical tissue from women who had undergone hysterectomies, to mimic physiological *in vivo* infection via the sexual contact route to extend our knowledge beyond more simple cell-cell infection studies; readily available tissue would be beneficial.

We utilised published protocols to establish an *ex vivo* organo-typical model for cervical tissue explants [3] aiming to co-culture HTLV-1 with live cervical explants. However, to facilitate reliable and reproducible studies of tissue co-cultures from the same donor at convenient time points and for purposes of long term storage of tissue for bio-banking, we sought to establish a protocol for cervical tissue cryopreservation akin to those validated for the bio-banking of other tissues, such as tonsils [11]. Here, we demonstrate that using the proposed protocol, cryopreserved cervical tissue can be thawed with minimal loss of tissue viability compared to fresh explants. We believe the development of this reproducible methodology permits long-term cervical tissue storage, thus reducing waste of precious tissue donations and, with relevant ethical approval in place, allows for cervical tissue bio-banking and tissue exploitation for a variety of studies.

## 2 Materials and methods

### 2.1 Ethics and tissue preparation

UK National Research Ethics Service (NRES 11/YH/0321), Research & Development at York Teaching Hospital NHS foundation Trust (YORA01992) and University of York ethically approved the study of *ex vivo* HTLV-1 cervical explant co-culture studies. Written informed consent was obtained from women with normal cervical smears who needed to undergo a planned hysterectomy for their own health and who were willing to donate cervical tissue. After the hysterectomy the uterus was transported to the histopathology department and reviewed macroscopically by the consultant histopathologist. Approximately 90% of the healthy cervix was released for research purposes.

Cervical tissue was immediately transferred into transportation medium (Leibovitz's L-15 medium containing heat-inactivated single-batch 10% foetal bovine serum (FBS), 100 U/ml penicillin, 100 μg/ml streptomycin, and 2.5 μg/ml amphotericin B [all from Invitrogen]) and cooled at 4 °C for transportation to research laboratories. All samples underwent processing within 4 h of surgery. Endo- and ecto-cervix were separated and all tissues were cut into approximately 1 cm^3^ explants containing mucosal and submucosal tissue to maintain tissue architecture. Tissue explants were either immediately processed for laboratory studies and tissue viability assays on explant pieces defined as day 0 (d0), or cryopreserved for subsequent testing.

### 2.2 Tissue freezing

Cervical explants (∼1 cm^3^) that were not immediately studied were placed individually into 2 ml cryovials on ice containing 1 ml of pre-cooled (4 °C) freezing medium (90% FBS with 10% dimethyl sulfoxide (Me_2_SO)) that was displaced by the explant ensuring cryoprotectant reached all the tissue. Cryovials were rapidly transferred to a control rate freezer pre-cooled to 4 °C (Planer KRYO560-16, Planer PLC, Sunbury-on-Thames, UK) and explants were cooled from 4 °C to −50 °C at 1 °C/min and then from −50 °C to −120 °C at 10 °C/min before transfer to liquid nitrogen for long term cryopreservation and bio-banking. Freezer chamber temperature data was recorded as evidence of successful cycle completion.

### 2.3 Tissue thawing

For this study, cervical explants were banked for an average of 189 days (range 119–236 days) before frozen tissue was thawed by cryovial retrieval from liquid nitrogen and immediate immersion in a water bath at 37 °C. As soon as freezing medium had started to thaw, the explant and thawed freezing medium was transferred, using forceps if necessary, to be completely submerged in 15 ml of pre-warmed (37 °C) culture medium (Roswell Park Memorial Institute 1640 medium containing heat-inactivated single-batch 10% FBS, 100 U/ml penicillin, 100 μg/ml streptomycin, all from Invitrogen) in 6-well plates in a sterile environment. Each explant was left undisturbed in this medium for 10 min at 37 °C in a humidified environment supplied with 5% CO_2_ in air before three further successive transfers to 15 ml of fresh culture medium, incubating for 10 min in each aliquot of 15 ml medium for a total time of 40 min. Tissue viability testing was immediately performed on explant pieces and defined as d0.

### 2.4 Tissue viability testing

We tested the viability of fresh and frozen-thawed cervical explants on the defined day 0 and after 1, 7 and 14 days in culture. Each cervical explant was cut up into at least 12 roughly equal-sized small pieces; individual explant pieces were transferred to single wells on a 96 well plate and covered with 200 μl culture medium before immediate testing or culture. Medium was changed every 3 days by removal of 170 μl of medium and replacement with 200 μl of fresh culture medium; empty wells were filled with medium to reduce evaporation. An MTT assay was used to establish general tissue viability by measuring the intensity of purple colour produced through mitochondrial oxidoreductase reduction of the MTT tetrazolium dye to its insoluble formazan, as described previously [14]. A 50 ml solution of Thiazolyl Blue Tetrazolium Bromide (Sigma-Aldrich, UK) [MTT] was prepared at 250 μg/ml in Roswell Park Memorial Institute 1640 medium and filtered through a 0.2 μm filter; 1 ml per well of this solution was aliquoted into three wells of a 48 well plate at each time point. A piece of explant was incubated with the 1 ml of MTT for 3 h under standard tissue culture conditions, in triplicate. After this incubation, tissue was transferred to 1 ml of methanol for dye elution and incubated for 16 h. Explants were removed and left to dry for 24 h, to increase accuracy, before weighing. Two-hundred μl of the tissue eluate in methanol was transferred in triplicate to a 96 well plate before optical density (OD) readings were taken at 595 nm (VersaMax plate reader, Molecular Devices, Wokingham, UK). Triplicate OD readings for methanol alone were made in parallel before subtraction from each sample OD followed by averaging of the triplicate readings for each piece of tissue. Tissue pieces were dried overnight before weight corrected OD (wcOD) was calculated using the formula: average OD reading/dried tissue weight (grams). The percentage metabolic activity as a measure of tissue viability was calculated by dividing the wcOD of each sample at each time point (day 0, 1, 7 and 14) by the averaged wcOD of triplicate pieces of tissue at day 0. We compared the wcOD of 10 fresh and three freeze-thawed tissues at day 0, 1, 7 and 14 to establish a quality control protocol for the cryopreservation of cervical tissue.

### 2.5 Explant sectioning and staining

Cervical tissue (fresh or freeze-thawed) after 1 day in culture was snap frozen in O·C.T. (Agar Scientific, Stansted, UK) on specimen discs (Leica, Loughborough, UK) seated on dry-ice then transferred to a −80 °C freezer. The blocks were sectioned (5 μm sections) using an OTF5000 cryostat microtome (Bright Instruments Ltd. Luton, UK), and applied to poly-lysine coated glass slides (Fisher Scientific, Loughborough, UK). Air-dried sections were haematoxylin and eosin (H&E) stained by immersing into filtered Mayers haematoxylin (Sigma-Aldrich, Poole, UK) for 5 min then rinsed in running tap water for an additional 5 min before dipping twelve times in 0.5% Eosin (Sigma-Aldrich). Sections were washed again in distilled water then dehydrated by dipping in sequential ethanol baths (50%, 70%, 95% and 100% EtOH) for between 30 and 60 s in each bath. Slides were air-dried and mounted using minimal DePeX (Sigma-Aldrich), a toluene-xylene-based mounting compound, and a coverslip. Sections were tile-scan imaged using a 10× objective on an Olympus B×51 microscope and MagnaFire SP software (Olympus, Southend-on-Sea, UK) before composite images were constructed in Photoshop (Adobe, Maidenhead, UK).

## 3 Results

From 19 separate cervical donations we were able to obtain, on average, 14 ± 6 explants of cervical tissue measuring approximately 1 cm^3^ that was identified by morphology as 1 ± 2 explants of endocervix and 13 ± 6 ectocervix explants (Supplementary Table 1). Explants surplus to experimental requirements were prepared and frozen as outlined and data recordings from the control rate freezer showed that the freezing process proceeded uninterrupted (data not shown). Using the protocols detailed above we performed viability assays at d0 on all cervical donations as well as on three randomly selected frozen/thawed-cervical tissue explants. These explants had been frozen for a mean of 189 days (range 119–236 days) and were thawed as described to coincide with the availability of a freshly harvested cervical explant. Viability assays were performed on pieces of the thawed explants and pieces of the fresh explant at this day 0 time point. Explant piece sizes were not significantly different in size (P > 0.05 using *t*-test), with fresh explants having a mean piece weight of 7.8 ± 0.7 mg and frozen-thawed pieces being 5.2 ± 0.4 mg. Remaining pieces of the same explants were then cultured in triplicate for 1, 7 and 14 days for additional viability assays. At all time points, macroscopically, both fresh and frozen tissue appeared similarly viable with both developing a purple colouring after incubation with the MTT reagent.

**Fig. 1:**
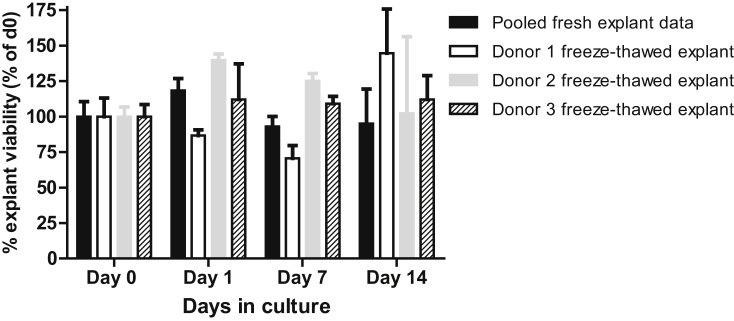
**MTT assay comparison of fresh and freeze-thawed cervical tissue explant viability.** The mean viability of seven fresh cervical explants (each performed in triplicate at every time point) compared to that of freeze-thawed cervical explants from three separate donors (in triplicate) at days 0, 1, 7 and 14 days assessed by MTT assay is shown. Average viability over time is expressed as a percentage of the respective explant's viability at day 0 ± SEM.

The mean optical density at day 0 of ten randomly selected fresh explants after weight correction was pooled and found to be 78.39 ± 11.79. The mean wcOD of the control fresh tissue used for this direct comparison study was 80.27 ± 9.80 and the three frozen-thawed triplicated explants had a wcOD of 82.60 ± 7.80 (Supplementary Table 2). No significant difference (P = 0.29) in metabolic activity (wcOD) as a measure of tissue viability was detectable between the pooled fresh- and thawed-explants at d0, using a *t*-test. Fig. 1 shows the viability of the three thawed explants compared to seven fresh samples during subsequent culture at days 1, 7 and 14; data is expressed as a percentage of the respective explant's wcOD at day 0. As can been observed, the viability of only one of the freeze-thawed explants was noticeably decreased from fresh tissue at days 1 and 7 whilst at day 14 even explant from donor 1 had similar viability to the fresh tissue (Fig. 1). No significant differences were found between the pooled tissue viability at d0 and the frozen-thawed tissue viability at d1, d7 or d14 using one-way ANOVA and Dunnett's Multiple Comparison Test.

**Fig. 2:**
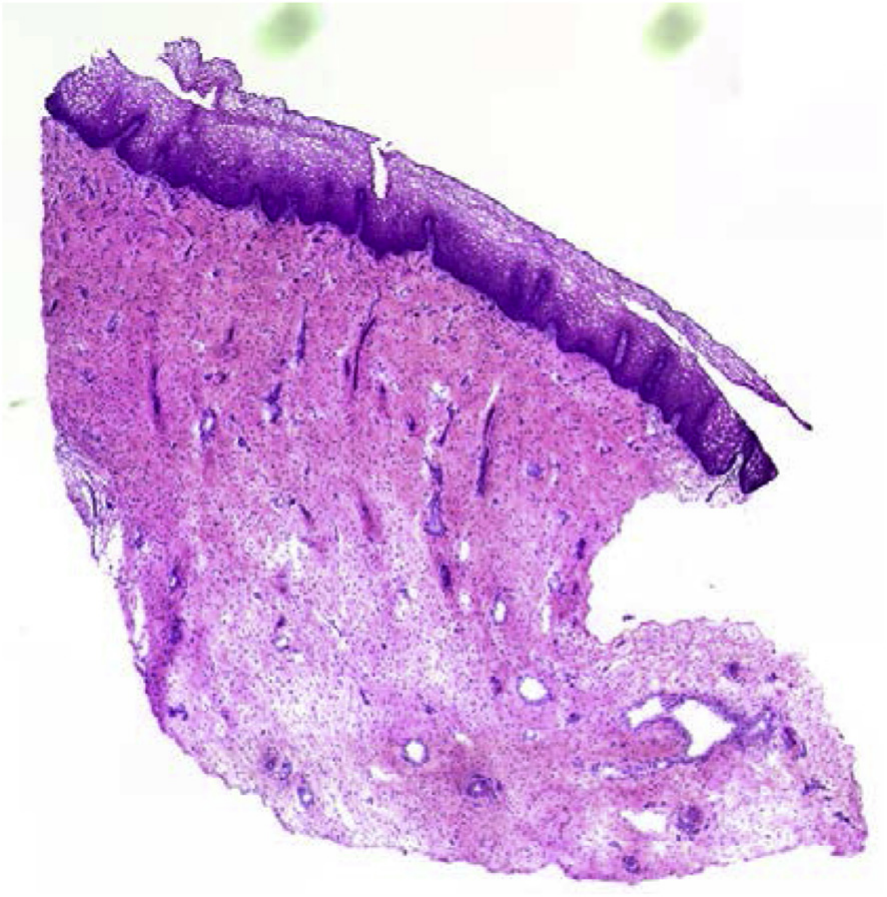
**Representative cervical tissue H&E staining of freeze-thawed cervical tissue.** Composite representation of sequential images of an H&E stained section of cervical tissue that had been frozen, cryopreserved, thawed then cultured to d1.

Furthermore, cervical explant pieces were sectioned, H&E stained and imaged to demonstrate that both frozen-thawed (Fig. 2) and fresh (Supplementary Fig. 1) tissue had acceptable morphology and had not been affected by tissue processing for culture and cutting into 1 cm^3^ explants for convenient storage in our bio-banking freezing procedure and subsequent thawing. The two images are comparable, although the eosin stain is a little more intense in the image of frozen-thawed tissue, clearly demonstrating that frozen-thawed tissue appears just as viable as fresh tissue.

## 4 Discussion

The rationale for this study was our desire to perform viral infection studies using *ex vivo* human cervical tissue replicating *in vivo* HTLV infections, which required the establishment of an organo-typical model. In order to conduct laboratory studies at convenience without losing previous cervical tissue we needed to store tissue long-term without a significant detrimental effect on tissue viability. Herein we describe the methodology used for the preparation, cryopreservation, bio-banking and thawing of cervical explants, which results in metabolically viable and morphologically intact tissue amenable for further experimentation. Our protocol is reliable and reproducible in preserving tissue viability.

Cervical tissue freezing and viable recovery has seldom been described in detail though Gupta et al., briefly communicated a method during their development of a cervical tissue-derived organ culture model [4]. Gupta and colleagues, using haematoxylin and eosin staining, showed that fresh as well as frozen-thawed cervical tissue maintained its integrity. In addition, they showed that there were no significant differences in the expression levels of cytokeratin and Ki67, two non-immune cellular markers, using immunohistochemistry. Furthermore, they demonstrated that both fresh and freeze-thawed tissue supported HIV-1 transmission and were equally useful for testing antiretroviral compounds. Our studies of tissue viability complement these findings and add important additional evidence, in addition to more comprehensive and reproducible methodology.

The major methodological differences between our approach and the study of Gupta et al., are that Gupta and colleagues examined cervical tissue that had been snap frozen in liquid nitrogen and stored at −80 °C using methodology described by the research group on a previous occasion [13]. Conversely to Gupta et al., in this study tissue was divided into 1 cm^3^ explants for convenient storage in cryovials, which had no significant deleterious effects on tissue viability or tissue integrity. We believe segmenting donated cervical tissue for long-term storage or bio-banking will prove more efficient than whole tissue storage since individual explants (an average of 14 × 1 cm^3^ explants can be obtained from representative donations) rather than the whole tissue can be thawed for future studies.

We used a slow freezing method, employed Me_2_SO as a cryoprotective agent (CPA) and stored our frozen cervical tissue in liquid nitrogen (approximately −196 °C) as opposed to the snap freezing procedures and storage at −80 °C described by previously [13]. We favoured this slow cryopreservation since we wished to store small segments of cervical donation and determined that the smaller tissue size would be suitable for the slow freezing process favoured for the cryopreservation of cell lines [5], where the employment of a freezing rate of 1 °C/min to −50 °C then 10 °C/min to −120 °C has been demonstrated to be the optimal cooling rate for mammalian cells [7]. We employed an active freezing approach through the utilisation of a control-rate freezer as this produces a more accurate and reproducible freezing rate that can be calibrated and freezing data can be recorded to ensure it has proceeded accordingly. This allowed recordability and afforded equipment calibration, which are important requirements for good laboratory practice and efficient bio-banking.

Due to its rapid permeability we used Me_2_SO as our CPA to protect cervical explant cells against intracellular and extracellular ice crystal formation during freezing [9]. We used 10% Me_2_SO in FBS that, although debatable, has previously been shown to have low toxicity [1]. The FBS that was used in our study was, for experimental consistency, heat-inactivated and from a single batch. Finally, we chose to store the frozen cervical explants at −196 °C in liquid nitrogen because this temperature provides greater stability to tissue biospecimens than storage at higher temperatures, likely due to the glass transition temperature of water being −135 °C [7].

Whilst previously reported cryopreservation methods are effective [4], [13], in our opinion, neither study provides sufficient detail for straightforward replication. Also, it is not clear how long their tissue had been frozen before it was thawed again, which made it difficult to assess any effect of long-term cryopreservation. The additional benefit of this study is therefore the provision of a comprehensive methodology for cervical tissue freezing and thawing and demonstrating that cervical tissue can be frozen for at least 236 days without any noticeable loss of tissue viability. Our study also provides useful details that, to our knowledge, have not been mentioned elsewhere, such as the number and amount of tissue that is likely to be available from cervical tissue donations (Supplementary Table 1).

Study limitations are that we didn't directly compare different methods of freezing and thawing. We considered that it would be ethically difficult to rationalise the additional usage of precious tissue having found a methodology that had little impact on tissue viability and architecture. Scientifically it would be interesting to directly compare the effects of different lengths of time from harvest to processing, different lengths of freezing, slow-versus snap-freezing, various CPAs and active versus passive freezing on the viability and integrity of stored cervical tissue. Active freezing processes may not be available to all laboratories; passive freezing approaches utilise economical, commercially available freezing containers filled with isopropanol, such as a Mr Frosty freezing container. These devices implement a cooling rate of 1 °C per minute when placed in a −80 °C freezer. If the device is left undisturbed and the isopropanol is changed frequently to avoid loss of alcohol through evaporation, as per manufacturer guidelines, passive freezing may be employed in the absence of a control-rate freezer. Janz Fde et al., reported that both approaches display similar potential to maintain phenotype, viability and function of certain tissue [8]. Finally, our methodology may be unsuitable for whole tissue freezing of cervical tissue should a larger sized sample be required.

To conclude, long-term cryopreservation of cervical explants is important to reduce tissue wastage and help with convenient planning of laboratory research without affecting research quality and to allow the preservation of surplus tissue for long-term bio-banking for future ethically approved *ex vivo* cervical explant studies. Herein we provide a detailed and extensive reproducible methodology to support this goal.

## Author disclosure statement

The authors have no institutional or commercial affiliations that might pose a conflict of interest regarding the publication of this study.

## Author contributions

JF and FM were responsible for designing the research project. FM secured the grant funding and national and local ethical approval for the project. AA, as the consultant histopathologist, reviewed and released cervical tissue under ethical approval to FM's study. CB was responsible for donor recruitment and administration of the study. JF and RW collected tissue, JF, RW and FM prepared and cryopreserved it. JF undertook the viability experiments described and JM performed the tissue sectioning and H&E staining. JF, RW and FM wrote the manuscript; all authors approved the final version.
