# Corrigendum to “Methodology for reliable and reproducible cryopreservation of human cervical tissue” [Cryobiology 77 (August) (2017) 14–18]

**DOI:** 10.1016/j.cryobiol.2017.08.005

**Published:** 2017-12

**Authors:** James M. Fox, Rebecca C. Wiggins, John W.J. Moore, Christine Brewer, Bill Hunter, Alison C. Andrew, Fabiola Martin

**Affiliations:** a Centre for Immunology and Infection, Department of Biology, Hull York Medical School, University of York, UK; b York Teaching Hospital, NHS Foundation Trust, York, UK

## Author contributions

JF and FM were responsible for designing the research project. FM secured the grant funding and national and local ethical approval for the project. **BH, as the consultant gynaecologist, consented patients and conducted the hysterectomies.** AA, as the consultant histopathologist, reviewed and released cervical tissue under ethical approval to FM's study. CB was responsible for donor recruitment, consenting and clinical administration of the study. JF and RW collected tissue, JF, RW and FM prepared and cryopreserved it. JF undertook the viability experiments described and JM performed the tissue sectioning and H&E staining. JF, RW and FM wrote the manuscript; all authors approved the final version.

Mr Bill Hunter was mistakenly omitted from the original version of the manuscript; this corrigendum rectifies this error.

The authors would like to apologize for any inconvenience caused.

DOI of original article: https://doi.org/10.1016/j.cryobiol.2017.06.004.

