# Supplementary material for: Methodology for reliable and reproducible cryopreservation of human cervical tissue
Source: Cryobiology. 2017 Aug;77:14–8. doi: 10.1016/j.cryobiol.2017.06.004 (PMC5536152; doi:10.1016/j.cryobiol.2017.06.004)
Supplement: Online data [file mmc1.docx]

**Supplementary Information**


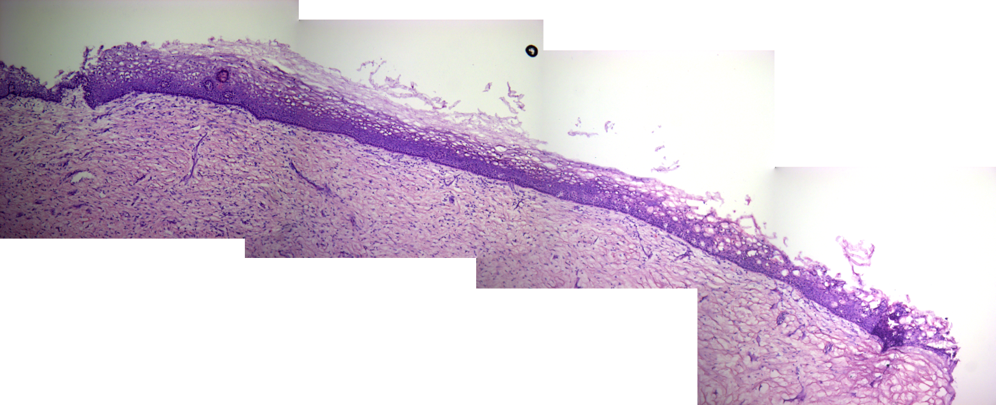


**Supplementary Figure 1 (relates to Figure 2). Representative cervical tissue H&E staining of fresh cervical tissue.** Composite representation of sequential images of an H&E stained section of d1 cultured fresh, i.e. non-cryopreserved, cervical tissue.

**Supplementary Table 1. Number of explants obtainable from nineteen cervical tissue donations**

| **Donor** | **Endocervical explants** | **Ectocervical explants** | **Total explants** |
| --- | --- | --- | --- |
| 1 | 6 | 3 | 9 |
| 2 | 0 | 9 | 9 |
| 3 | 0 | 1 | 1 |
| 4 | 0 | 3 | 3 |
| 5 | 0 | 17 | 17 |
| 6 | 4 | 15 | 19 |
| 7 | 4 | 14 | 18 |
| 8 | 0 | 18 | 18 |
| 9 | 0 | 15 | 15 |
| 10 | 0 | 15 | 15 |
| 11 | 0 | 14 | 14 |
| 12 | 4 | 15 | 19 |
| 13 | 0 | 9 | 9 |
| 14 | 0 | 11 | 11 |
| 15 | 0 | 15 | 15 |
| 16 | 0 | 18 | 18 |
| 17 | 0 | 18 | 18 |
| 18 | 0 | 20 | 20 |
| 19 | 0 | 24 | 24 |
| **Mean** | **1** | **13** | **14** |
| **Standard deviation** | **2** | **6** | **6** |

**Supplementary Table 2. Weight corrected optical density at d0 of explants from ten freshly processed cervical tissue donations and of three frozen/thawed explants from individual donors**

|  | **Fresh** | **Frozen/thawed** |
| --- | --- | --- |
|  | 174.28 | 88.87 |
|  | 83.54 | 67.10 |
|  | 75.20 | 91.82 |
|  | 60.37 |  |
|  | 53.90 |  |
|  | 80.27 |  |
|  | 78.00 |  |
|  | 58.22 |  |
|  | 34.85 |  |
|  | 85.26 |  |
| **Mean** | **78.39** | **82.60** |
| **Standard error** | **11.79** | **7.80** |
